# Prognostic value of respiratory compliance course on mortality in COVID-19 patients with vv-ECMO

**DOI:** 10.1186/s13613-023-01152-7

**Published:** 2023-06-21

**Authors:** Simon Valentin, Mathieu Amalric, Guillaume Granier, Benjamin Pequignot, Christophe Guervilly, Kevin Duarte, Nicolas Girerd, Bruno Levy, Paul Dunand, Matthieu Koszutski, Hadrien Roze, Antoine Kimmoun

**Affiliations:** 1 CHRU de Nancy, Médecine Intensive et Réanimation Brabois, Université de Lorraine, Nancy, France; 2 CHRU de Nancy, Pôle des Spécialités Médicales/Département de Pneumologie, Université de Lorraine, Nancy, France; 3 INSERM U1254 IADI, Université de Lorraine, Nancy, France; 4 Médecine Intensive et Réanimation, Hôpital Nord, Assistance Publique - Hôpitaux de Marseille, Marseille, France; 5 Centre d’Etudes et de Recherches sur les Services de Santé et qualité de vie EA 3279, Faculté de Médecine, Aix-Marseille Université, Marseille, France; 6 INSERM U1116, Université de Lorraine, Nancy, France; 7 INSERM 1433 CIC-P CHRU de Nancy, FCRIN INI-CRCT, Université de Lorraine, Nancy, France; 8 Département d’anesthésie Réanimation Sud, Centre Médico-Chirurgical Magellan, Hôpital, Haut Leveque Hospital, Université de Bordeaux, Pessac, France; 9 INSERM 1045, Centre de Recherche Cardio Thoracique, Pessac, France

**Keywords:** Compliance, COVID-19, ECMO, Respiratory distress syndrome

## Abstract

**Background:**

COVID-19-associated acute respiratory distress syndrome (ARDS) supported by veno-venous extra-corporal membrane oxygenation (vv-ECMO) results in a high in-hospital mortality rate of more than 35%. However, after cannulation, no prognostic factor has been described to guide the management of these patients. The objective was to assess the association between static respiratory compliance over the first 10 days post-vv-ECMO implantation on 180-day mortality.

**Results:**

In this multicentric retrospective study in three ECMO referral centers, all patients with COVID-19-associated ARDS supported by vv-ECMO were included from 03/01/2020 to 12/31/2021. Patients were ventilated with ultra-protective settings targeting a driving pressure lower than 15 cmH_2_O. 122 patients were included. Median age was 59 IQR (52–64), 83 (68%) were male, with a median body mass index of 33 (28–37) kg/m^2^. Delay between first symptoms to vv-ECMO implantation was 16 (10–21) days. Six-month death was 48%. Over the first ten days, compliance increased in 180 day survivors [from 18 (12–25) to 20 (15–27) mL/cmH_2_O] compared to non-survivors [from 12 (9–20) to 10 (8–14) mL/cmH_2_O, *p* interaction < 0.0001]. A time varying multivariable Cox model found age, history of chronic lung disease, compliance from day one to day ten and sweep gas flow from day one to day ten as independent factors associated with 180-day mortality.

**Conclusions:**

In COVID-19-associated ARDS, static respiratory compliance course over the first ten days post-vv-ECMO implantation is associated with 180-day mortality. This new information may provide crucial information on the patient's prognosis for intensivists.

**Supplementary Information:**

The online version contains supplementary material available at 10.1186/s13613-023-01152-7.

## Key points

- Question: Does static respiratory compliance predict outcomes in patients with COVID-19 associated acute respiratory distress syndrome requiring vv-ECMO?
- Findings: In 122 included patients, static respiratory compliance course over the first ten days post vv-ECMO implantation was associated with 180-day mortality in a multicentric retrospective study.
- Meanings: Monitoring static respiratory compliance during the ten first days in these patients may provide crucial information on their prognosis and help intensivits for their management.

**Supplementary Information:**

The online version contains supplementary material available at 10.1186/s13613-023-01152-7.

## Background

Since the beginning of COVID-19 pandemic, the extracorporeal life support organization reported more than 10 500 patients with COVID-19-associated ARDS (CARDS) supported by vv-ECMO [1]. The in-hospital mortality of these patients has changed over time, initially from around 30% to reach more than 65% in last large series. These rates are no longer comparable to non-COVID vv-ECMO-treated patients [2–4]. Reasons of this increase remain widely misunderstood and are probably multifactorial with virus-related pathogenicity, vv-ECMO delay and patients being more comorbid [2]. Risk factors of mortality, such as a prolonged pre-ECMO duration of mechanical ventilation, have been identified in early studies, [5, 6]. These factors are used to select patients and once on vv-ECMO, they receive ultra-protective ventilation with limited driving pressure below 15 cmH_2_O and stable levels of positive end-expiratory pressure (PEEP) [4]. The severity of lung injury with the reduction of aerated lung is correlated to the static respiratory compliance (Crs), and this Crs can decrease significantly after ECMO start [4]. Lung function recovery may take longer time in patients with COVID-19 supported by vv-ECMO [7]. Evolution of early clinical parameters in a context of prolonged intensive care unit (ICU) stay durations and very high mortality under vv-ECMO might be useful hints for prognostic assessment. Indeed, therapeutic withdrawal can be a challenging task especially in a pandemic context during which the number of ICU beds could be limited [8].

To address this need, our hypothesis was that, in selected patients under vv-ECMO, the evolution of respiratory parameters in the first ten days following vv-ECMO implantation for CARDS might be associated with the outcome. Thus, the objective of this study was to assess the impact of Crs evolution in the first ten days under vv-ECMO on 180-day mortality.

## Methods

### Study design

This retrospective study was conducted in three referral ECMO university hospitals from March 1, 2020, to December 31, 2021, in France. The study was registered in clinical trial registry before the collection and the analysis of the data: https://www.clinicaltrials.gov/ct2/show/NCT05341687).

### Inclusion criteria

All consecutive adult patients hospitalized in one of the three ICU with a confirmed CARDS requiring vv-ECMO, were included.

### Pre-vv-ECMO management

Diagnosis of SARS-CoV-2 infection was made by a positive reverse transcriptase polymerase chain reaction (RT-PCR) on nasopharyngeal swab or lower respiratory tract sampling, and a chest computed tomography with typical abnormalities, as described by the *Fleischner Society* [9].

The decision to initiate vv-ECMO was systematically discussed by a multidisciplinary expert team, after optimization of the mechanical ventilatory support (V_T_ lower or equal to 6 mL/kg of ideal body weight, use of PEEP adjusted to target a plateau pressure lower or equal to 28–30 cmH_2_O), at least after one session of prone positioning (when technically possible) and neuro-muscular blocking agents (see Additional file).

Criteria for vv-ECMO implantation were based on the EOLIA inclusion criteria [10].

### Management after implantation of vv-ECMO

Management was homogenous among the centers, as all were participating in multicenter trials on vv-ECMO management such as PRONECMO (NCT04607551) or EOLIA trials [10].

#### General ventilatory support management

Immediately after vv-ECMO implantation, an ultra-protective lung ventilation to enhance ventilator-induced lung injuries prevention was started as proposed in the EOLIA trial [10]. Briefly, the ventilatory mode could be either volume- or pressure-control. For both ventilatory modes, as a general guideline, intensivists were encouraged to maintain ΔP below 15 cmH_2_O and to adapt ventilatory parameters accordingly. Respiratory rate (RR) was reduced to 8–12 c/min. In volume control mode, *V*_T_ was reduced to 2–3 mL/kg of ideal body weight, PEEP was generally maintained above 10 cmH_2_O with a plateau pressure (P_plat_) that should not exceed 23 to 25 cmH_2_O. In pressure-control mode, high pressure (P_high_) and low pressure (P_low_) were set between 23 and 25 cmH_2_O and above 10 cmH_2_O, respectively.

#### vv-ECMO settings

ECMO blood flow was set to target an arterial oxyhaemoglobin saturation (SaO_2_) above 90%. Beta blockers, deep sedation, or moderate hypothermia applied with ECMO-heat exchanger could be used to reduce the cardiac output when SaO_2_ remained below 90% with maximal ECMO blood flow [11]. Sweep gas flow was started at 1 L/min and increased slowly to avoid a rapid drop in PaCO_2_ and then adapted to ideally reach a pH between 7.38 and 7.42 mmHg. ECMO membrane efficiency was checked daily by the perfusionist.

#### Corticosteroids management

After the release of the recovery trial in June 2020, all patients were early treated by dexamethasone at a posology of 6 mg/day for a total duration of 10 days [12]. Thereafter, in case of non-clinical improvement after vv-ECMO implantation, a corticosteroid treatment could be initiated at physician discretions [13].

#### Prone positioning

Prone positioning in patients undergoing vv-ECMO was left at the physician discretion until April 1, 2021. Thereafter, a part of patients undergoing vv-ECMO were included in the *proneECMO* trial in which the decision to prone a patient was randomized (NCT04607551).

Details of management, including withdrawal of care are detailed in the additional file online.

### Main outcomes

The primary outcome of this study was to assess, in patients with CARDS, the association between the Crs of the first ten days after vv-ECMO implantation and 180 day mortality.

Secondary outcome was to describe the evolution of the other ventilatory parameters over the first 10 days (PEEP, *V*_T_, ΔP, RR, P_plat,_ ECMO blood and sweep gas flows).

### Data collection

Baseline time was defined as the first day after vv-ECMO implantation. Demographics, medical history, SAPS II score, timing of first respiratory symptoms, ICU admission, endotracheal intubation and vv-ECMO implantation were recorded. Waves of pandemic were defined according to official dates provided by the French National Institute for Statistics and Economic Studies (www.insee.fr). Ventilatory parameters were collected just before vv-ECMO implantation and then daily from day one to day ten and included ventilator mode and settings (*i.e.*, RR, PEEP, P_plat_, ΔP, Crs). ΔP was defined as P_plat_ minus total PEEP. vv-ECMO settings (*i.e.*, vv-ECMO blood flow, sweep gas flow) were also recorded daily from day one to day ten. Arterial blood gases were collected just before vv-ECMO implantation and then at day one, two and ten. In patients ventilated with pressure control mode, P_high_ was considered as P_plat_ and P_low_ as PEEP. Ventilatory acquired pneumonia or sepsis before vv-ECMO implantation was defined as a documented pneumonia occurring more than 48 h after mechanical ventilation start or as the initiation of an antibiotic therapy. Dates of explantation from vv-ECMO, weaning from mechanical ventilation (MV), ICU discharge and death until 180 days after ICU admission were recorded. Vital status at day 180 was assessed using the national open-access database matchID (https://deces.matchid.io/).

### Ethics

In accordance with French legislation, non-opposition of the patient or their legal representative for use of the data was systematically sought. The study was approved by the ethical committee of Nancy teaching Hospital (N°CO-26). All data were collected into an anonymous computerized database and registered to the electronic data registry of Nancy teaching hospital (N°2022PI050-235). The procedures followed were in accordance with the ethical standards of the responsible committee on human experimentation and with the Helsinki Declaration of 1975, as most recently amended.

### Statistical analysis

Analytical data are presented as the median with 25th and 75th percentiles [median (interquartile range)] for continuous variables, whereas categorical variables as numbers and percentages. Comparisons of baseline characteristics according to waves of the pandemic were conducted by using Wilcoxon or Kruskal–Wallis tests for continuous variables and the Fisher exact test or *χ*2 test for categorical variables. Comparisons of characteristics before and after vv-ECMO implantation were handled with a Wilcoxon rank signed test. Continuous variables analyzed each day from day one to day ten were handled by a linear mixed model tested with Kenward–Roger’s F tests. Trend in proportion evolutions over time were assessed using a Cochran–Armitage Q test.

The primary outcome measure was the prevalence of 180 day mortality after ICU admission. To determine factors associated with 180 day mortality while considering the changes in mechanical ventilatory parameter measurements over time, a Cox model with time-dependent covariates was performed (see the additional file online for explanation on time-dependent Cox regression method). For illustration purpose, a survival curve by using the Kaplan–Meier method was drawn according to the Cox model with Crs as a time-dependent covariate. Groups in this figure were based on terciles of all Crs values from day one to ten. The results were presented as hazard ratio with 2.5% and 97.5% values. Considering that there are 8.7% missing data for variables included in the multivariable model, an imputation analysis has been performed using simple two-stage approach (see details in Additional file 1: Fig. S1) [14]. Additional statistical analyses to assess the best thresholds (according to ROC curves and Cox regression analysis) of delta Crs between day one to day five or to day ten were performed with calculation of sensitivity and specificity and C-index for each threshold. A two-sided *p*-value ≤ 0.05 was regarded as statistically significant. Statistical analyses were performed using R, version 4.1.1 (2021-08-10) (R Foundation for Statistical Computing, Vienna, Austria).

## Results

### Description of the population at baseline

One hundred twenty-two consecutive patients were hospitalized for COVID-19 requiring vv-ECMO from March 1, 2020, to December 31, 2021. Baseline characteristics are presented in Table 1. Briefly, median age was 59 (52–64) years, 83 (68%) were male, with a median BMI of 33 (28–37) kg/m^2^ and most of them, *i.e*., 53 (43%), were included during the third wave.

**Table 1:** Description of baseline characteristics according to 180-day outcome

Variable	*N*	Total population	*N*	Survivors	*N*	Non-survivors	*p*
Demographics							
Age (years)	122	59 (52–64)	64	56 (46–62)	58	62 (58–66)	< 0.0001
Male gender (%)	122	83 (68%)	64	41 (64%)	58	42 (72%)	0.32
Body mass index (Kg/m^2^)	122	33 (28–37)	64	34 (29–40)	58	32 (27–36)	0.13
Medical history							
Hypertension	122	60 (49%)	64	31 (48%)	58	29 (50%)	0.86
Diabetes mellitus	122	30 (25%)	64	12 (19%)	58	18 (31%)	0.12
Cardiac disease	122	11 (9%)	64	5 (8%)	58	6 (10%)	0.63
Lung disease	122	29 (24%)	64	11 (17%)	58	18 (31%)	0.073
Renal disease	122	10 (8%)	64	6 (9%)	58	4 (7%)	0.75
Immunosuppression	122	15 (12%)	64	8 (12%)	58	7 (12%)	0.94
Wave of pandemic	122		64		58		0.009
Wave 1		23 (19%)		19 (30%)		4 (7%)	
Wave 2		25 (20%)		9 (14%)		16 (28%)	
Wave 3		53 (43%)		25 (39%)		28 (48%)	
Wave 4		21 (17%)		11 (17%)		10 (17%)	
Pre ECMO period							
SAPS2 score	122	43 (34–55)	64	42 (32–54)	58	43 (35–58)	0.27
Delay between first symptoms to ECMO (days)	109	16 (10–21)	62	13 (9—20)	47	17 (11–22)	0.070
Length of invasive mechanical ventilation (days)	122	4 (1–9)	64	3 (1–6)	58	6 (2–10)	0.011
Corticosteroids for COVID-19	122	85 (70%)	64	37 (58%)	58	48 (83%)	0.003
Corticosteroids for prolonged ARDS	122	82 (67%)	64	36 (56%)	58	46 (79%)	0.007
Tocilizumab for COVID-19	122	16 (13%)	64	6 (9%)	58	10 (17%)	0.20
Prone positioning	122	117 (96%)	64	60 (94%)	58	57 (98%)	0.37
Mobile ECMO assistance	122	67 (55%)	64	34 (53%)	58	33 (57%)	0.68
Ventilator acquired pneumonia	122	72 (59%)	64	31 (48%)	58	41 (71%)	0.013
Day 1 parameters							
Lactate (mmol/l)	119	1.4 (1.0–2.1)	64	1.4 (0.9–1.8)	55	1.5 (1.1–2.6)	0.24
PaO_2_/FiO_2_	122	134 (93–175)	64	138 (108–163)	58	124 (91–182)	0.55
Respiratory rate (c/min)	119	12 (10–15)	64	12 (10–15)	55	12 (10–12)	0.14
Tidal volume (mL/kg)	119	2.8 (2.0–3.5)	64	3.0 (2.4–3.8)	55	2.3 (1.8–3.2)	0.001
Static respiratory compliance (mL/cmH_2_O)	116	15 (10–22)	61	18 (12–25)	55	12 (9–20)	0.006
Plateau pressure (cmH_2_O)	114	24 (22–26)	60	24 (22–26)	54	25 (22–27)	0.49
PEEP (cmH_2_O)	121	12 (10–15)	64	12 (10–15)	57	12 (10–14)	0.53
Driving pressure (cmH_2_O)	114	12 (10–15)	60	12 (10–14)	54	12 (9–15)	0.46
Outcomes							
ECMO duration (days)	122	20 (11–30)	64	13 (9–22)	58	28 (19–33)	< 0.0001
Invasive mechanical ventilation duration (days)	122	33 (23–50)	64	30 (21–50)	58	38 (26–50)	0.17
Vasopressors (day one to day ten, yes/no)	122	83 (68%)	64	40 (62%)	58	43 (74%)	0.17
Number of days on vasopressors (day one to 10) 1 o	100	1 (0–4)	51	1 (0–4)	49	2 (0–3)	0.35
In-ICU length of stay (days)	122	38 (27–52)	64	37 (27–58)	58	39 (28–49)	0.81
Six-month deaths	122	58 (48%)	64	0 (0%)	58	58 (100%)	< 0.0001

Values are expressed as median [IQR] or as number and frequency. n: available data

*COVID-19* coronavirus disease 2019, *ECMO* extracorporeal membrane oxygenation, *FiO*_*2*_ inspired oxygen fraction, *ICU* intensive care unit, *PaO*_*2*_ arterial oxygen tension, *PEEP* positive end-expiratory pressure

Change in ventilatory parameter measurements before and after vv-ECMO initiation is presented in Additional file 1: Table S1. Initiation of vv-ECMO was associated with a reduction in *V*_T_ from 6 (5–6) mL/kg to 3 (2–4) mL/kg, *p* < 0.0001. ΔP decreased from 17 (15–20) to 12 (9–14) cmH_2_O, *p* < 0.0001 and RR from 28 (26–30) to 12 (10–15) c/min, *p* < 0.0001 and PEEP slightly increase from 12 (9–14) to 12 (10–15) cmH_2_O, *p* = 0.0116).

After vv-ECMO implantation, 34% of patients received pressure-controlled mode, and 66% remained in volume-controlled mode. Vv-ECMO duration was 20 (11–30) days, invasive MV duration was 33 (23–50) days and ICU length of stay was 38 (27–52) days. No patient presented a severe right ventricular dysfunction over the ten first days and none underwent a conversion from VV to VA of VVA ECMO settings.

Fifty-eight patients had died at 180 days. Compared to survivors, non-survivors were older 62 (58–66) *vs* 56 (46–62) years, (*p* < 0.0001), tended to have more chronic lung disease (*p* = 0.073), had a longer invasive MV duration before ECMO implantation [6 (2–10) *vs* 3 (1–6), *p* = 0.011]. V_T_ and Crs at day one were significantly different between non-survivors and survivors [2.3 (1.8–3.2) *vs* 3.0 (2.4–3.8) mL/kg, *p* = 0.001 and 12 (9–20) *vs* 18 (12–25) mL/cmH_2_O, *p* = 0.006, respectively]. Vv-ECMO duration was longer in non-survivors as compared with survivors [28 (19–33) *vs* 13 (9–22) days, *p* < 0.0001]. Among the 58 non-survivors, 35 had undergone a withdrawal of care, 22 developed a multiple organ failure and 1 died from ECMO-related fatal adverse event. A CT scan was performed right before death in 29/58 (50.0%) patients. Among them, 23 were carried out as part of a withdrawal of care process. Intra-lobular reticulations or traction bronchiectasis were found in 19/23 (82.6%). Evolution of baseline characteristics over the four waves of pandemic is described in Additional file 1: Table S2.

### Static respiratory compliance assessment over time

**Fig. 1:**
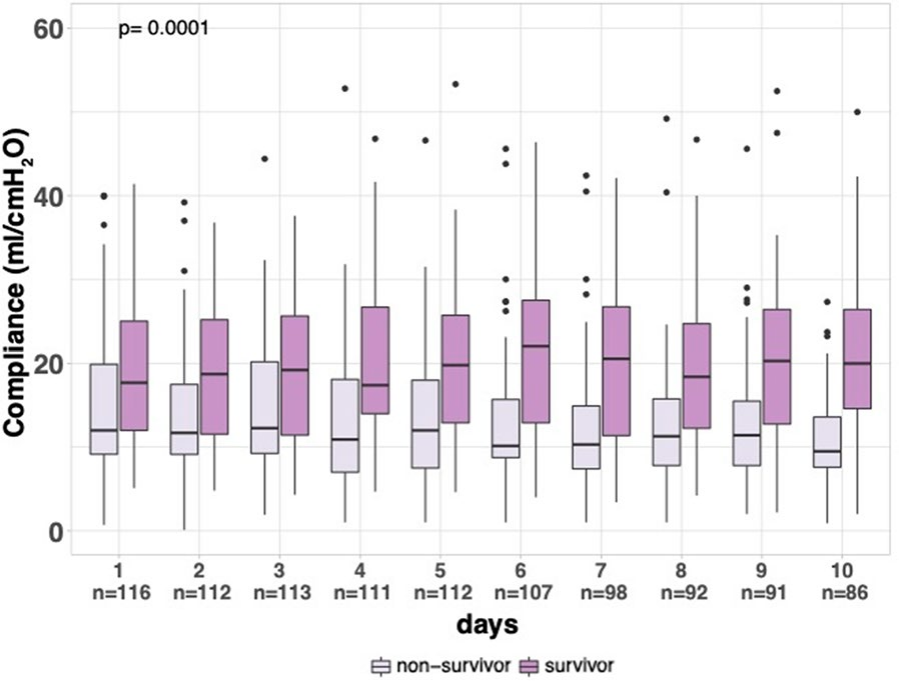
Static respiratory compliance over time according to 180 day status. Crs: static respiratory compliance in mL/cmH_2_O

Figure 1 shows the course of Crs according to 180-day vital status over the first ten days. Crs increased over the first ten days in 180 day survivors from 18 (12–25) to 20 (15–27) mL/cmH_2_O compared to non-survivors in which Crs decreased from 12 (9–20) to 10 (8–14) mL/cmH_2_O (*p* interaction = 0.0001).

**Fig. 2:**
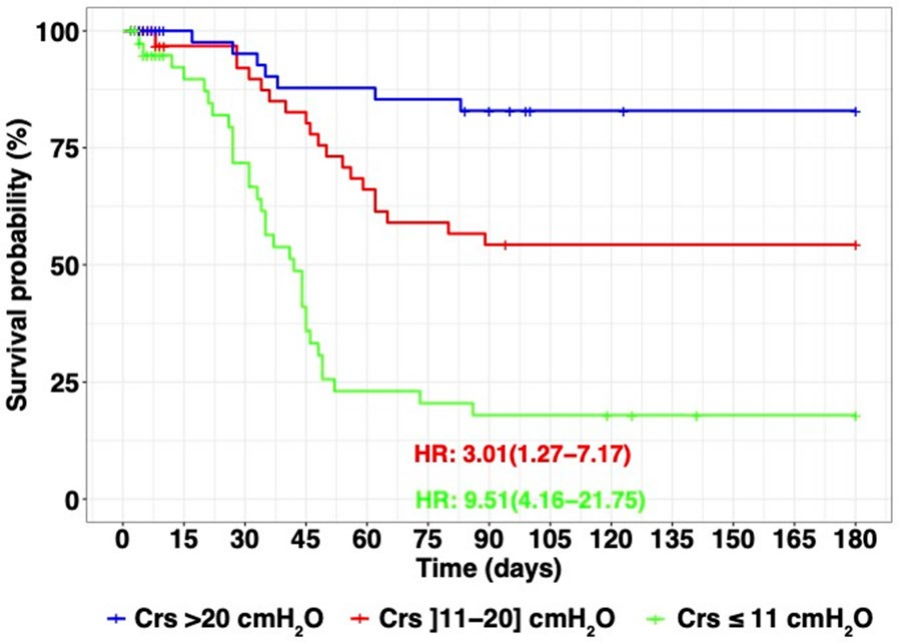
Kaplan–Meier curves with Crs as a time-dependant covariate and represented according to tercile of all Crs from day one to day ten. Reference group for univariate Cox model is Crs > 20 mL/cmH_2_O. *HR* hazard ratio

Among the 122 patients included 58 (48%) died within 180 days. Figure 2 shows the Kaplan–Meier curves according to terciles of all Crs recorded from day one to day ten post vv-ECMO implantation. Compared to patients in the third tercile of Crs > 20 mL/cmH_2_O, patients in the first (< 11 mL/cmH_2_O) and second tercile of Crs (from 11 to 20 mL/cmH_2_O) presented a higher risk of death with HR: 9.51 CI 95% (4.16 to 21.75) and 3.01 CI 95% (1.27 to 7.17), respectively.

### 180-day mortality predictors

Univariate Cox model with time-dependent and time-fixed variables are presented in Additional file 1: Table S3. Multivariate Cox model retained older age, history of chronic lung disease, and increase in sweep gas flow over time to be associated with a higher risk of 180-day mortality. The increase in Crs over time was associated with a better outcome (Table 2). Results were similar after multiple imputations.

**Table 2:** Predictors of 180-day mortality in patients with CARDS

Variables	HR 95% CI	*p*
Time-fixed variables		
Age (per 5 years)	1.38 (1.15–1.65)	0.0004
Chronic lung disease	1.86 (1.05–3.31)	0.0346
Time-dependent variables		
Static respiratory compliance (per 5 mL/cmH_2_O) from day 1 to 10	0.64 (0.52–0.79)	< 0.0001
Sweep gas flow (L/min) from day 1 to 10	1.16 (1.04–1.29)	0.0097

Best thresholds of delta Crs between day one to day five or to day ten have been evaluated. The best threshold obtained from the ROC curve for delta day 1 day 5 (*n* = 107 patients with complete data) was 3 (− 1—5) mL/cmH_2_O with an AUC at 0.61. Sensitivity was at 0.86 (0.57–0.98) and specificity at 0.46 (0.27–0.77). The best threshold obtained from the ROC curve for delta day 1 day 10 (*n* = 83 patients with complete data) was 4 (3–5) mL/cmH_2_O with an AUC at 0.68. Sensitivity was at 0.92 (0.81–0.98) and specificity at 0.54 (0.37–0.71). With a Cox regression method, similar thresholds were found (delta Crs day 1 day 5: 3 mL/cmH_2_O, C-index: 0.58; delta Crs day 1 day 10: 4 mL/cmH_2_O, C-index: 0.65).

### Other ventilatory parameters analysis over time

**Fig. 3:**
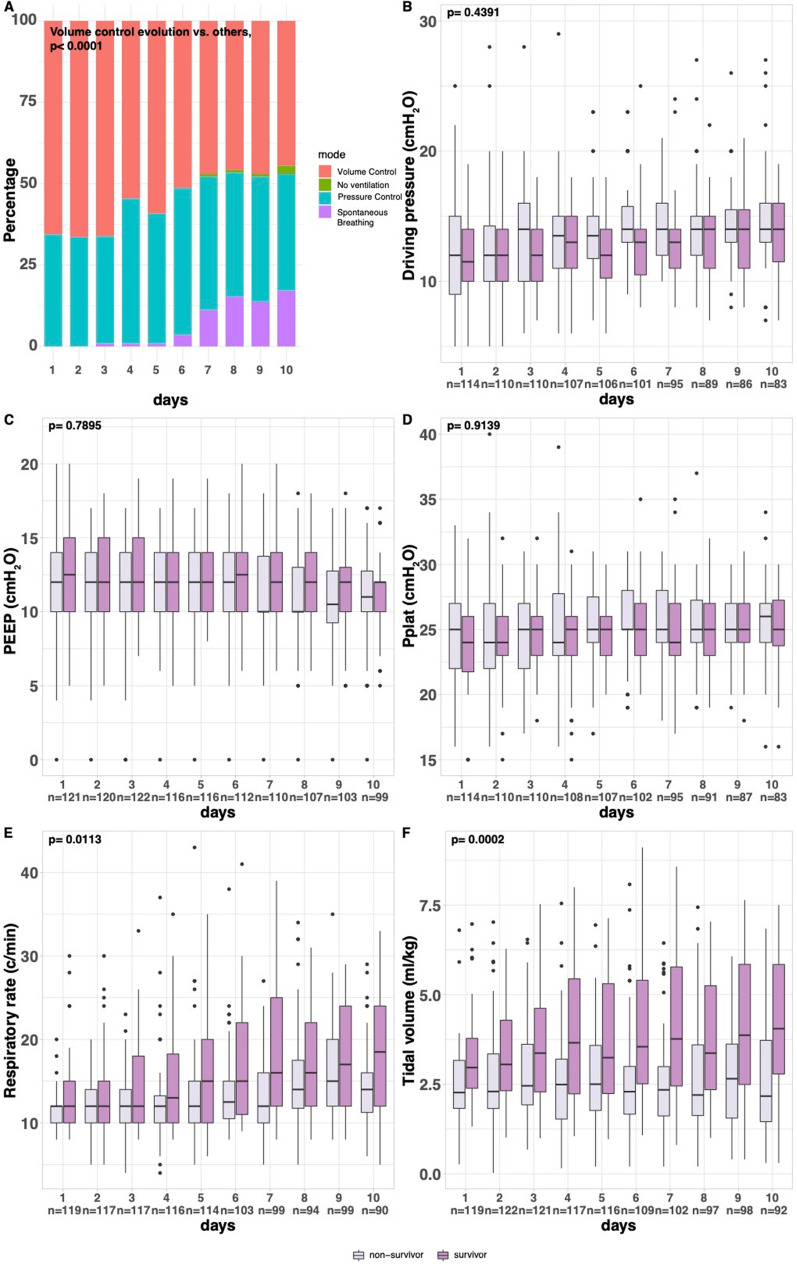
Exploratory ventilatory variables recorded daily from day one to day ten and according to 180 day status. Panel A: ventilatory modes. Panel B: driving pressure. Panel C: positive end-expiratory pressure. Panel D: plateau pressure. Panel E: respiratory rate. Panel F: tidal volume

Figure 3 shows the ventilatory parameters over the first ten days after vv-ECMO implantation.

Ventilatory modes were mainly “volume control” and “pressure control” with a decrease in proportions of volume control mode over the first ten days (*p* < 0.0001) (Fig. 3A). There were no interactions between time and 180-day outcome on $\Delta$ P, PEEP and P_plat_ ventilatory variables which remained all globally constant over time (Fig. 3B–D). By contrast, RR and V_T_ increased over time in 180-day survivors compared to 180-day non-survivors (*p* interaction = 0.0113 and p interaction = 0.0002, respectively, Fig. 3E, F).

**Fig. 4:**
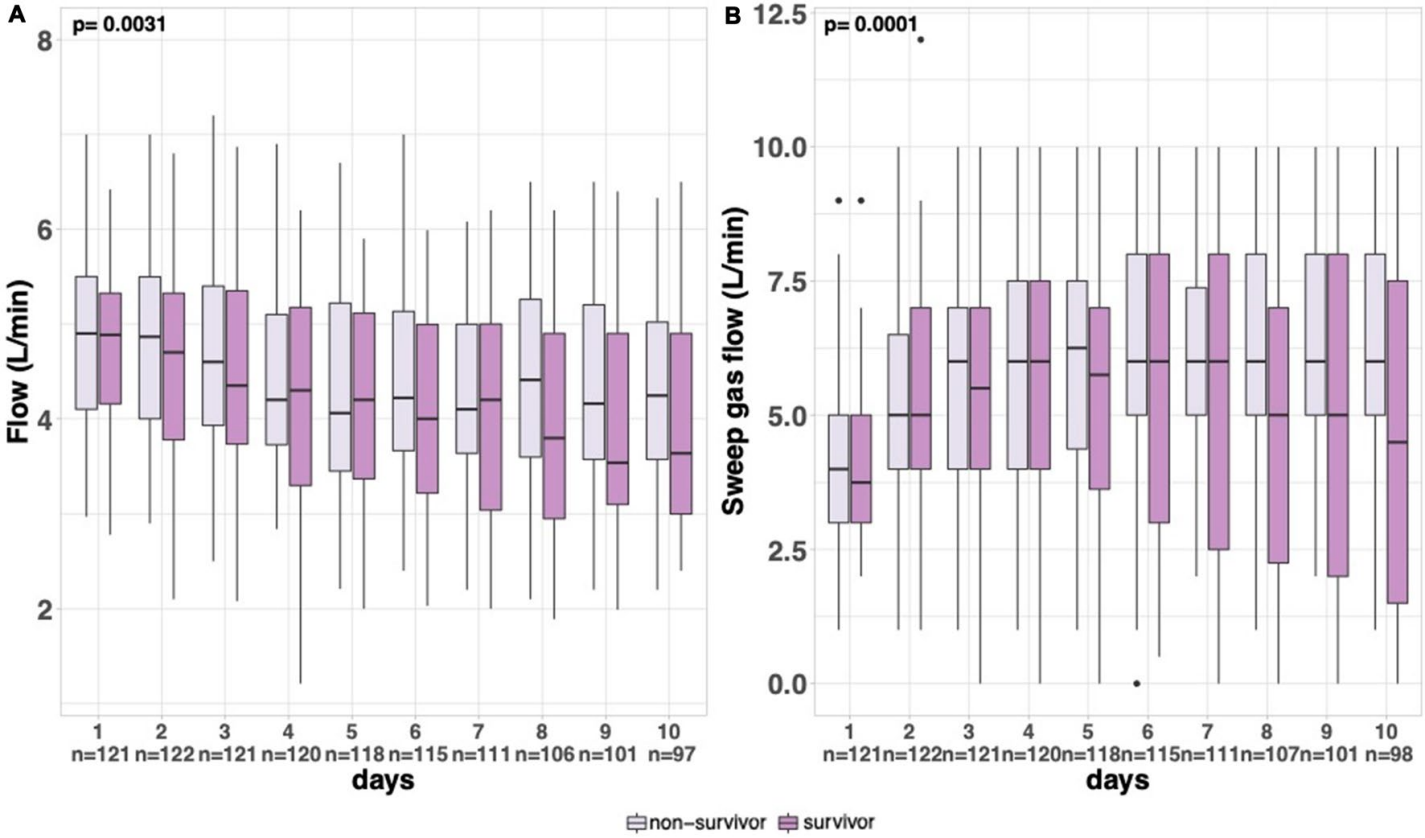
Exploratory vv-ECMO parameters recorded daily from day one to day ten and according to 180 day status. Panel A: vv-ECMO flow. Panel B: sweep gas flow

Figure 4 shows that vv-ECMO flow and sweep gas flow over the ten first days decreased more in survivors than in non-survivors (*p* interaction = 0.0031 and *p* = 0.0001, respectively, Fig. 4A, B).

## Discussion

The main result of the present study is that Crs evolution on the first ten days was independently associated with 180-day survival status. Importantly, all the included patients received protective low *V*_T_, low P_plat_, moderate to high PEEP and almost all had prone position session before vv-ECMO implantation. Finally, when undergoing vv-ECMO, “ultraprotective ventilation” was applied in most patients with a target of $\Delta$ P below 15 cmH_2_O.

The prognostic value of early Crs course after ECMO implantation remains debated specifically for CARDS on ECMO in whom respiratory evolution is delayed compared to non-COVID-19 ARDS on ECMO. No major recent study reported the association between early Crs evolution post-ECMO implantation and the outcome in CARDS patients [3, 6, 15–20]. In LIFEGARDS from Schmidt et al*.* which included only non-COVID-19 ARDS, driving pressure but not Crs over the first days has been reported associated with 180-day outcome [4].

The observed mortality (48%) was higher than previously reported. Schmidt et al.*,* first reported from March to May 2020, 83 cases of CARDS supported by vv-ECMO with a 60-day mortality of 31% [3]. Later, Nesseler et al*.* conducted a nationwide French study from March to October 2020 and observed a 28 day in-hospital mortality of 51% [6]. Several reasons could explain the difference in mortality observed. First, period of inclusion included different waves of pandemic. Patients, in the Schmidt et al*.,* study, were included only during the first wave of pandemic in which the mortality rate was lower as we also observed in the present study (17%) [3]. By contrast, the mortality rate in the Nesseler et al*.,* study was close to the global mortality rate observed in our study (48%). They included patients from the first two waves and patients from low, medium, and high-volume ECMO centers which may have impacted the outcome [6]. Finally, others also demonstrated that successive waves of pandemic were associated with increasing mortality rates [21, 22]. Explanations may be numerous, and our study includes older patients, tending to have more comorbidities but also being more severe on ventilatory parameters at day one.

The management of patients with ARDS before vv-ECMO has greatly improved over the last few years. Indeed, from only 26% of the patients being prone positioned in LIFEGUARD study to 56% in the ECMO group of the EOLIA study, 96% were prone positioned in the present study [4, 10]. Similarly, recommendations on protective mechanical ventilation in ARDS patients were also far better respected, whereas patients presented a critical respiratory state [23].

Our main result was that Crs evolution over the first ten days was associated with 180-day outcome. This finding may be a surrogate of lung injury recovery which occurs early only in survivors. Before ECMO, Crs was low with severe hypoxemia as a consequence of lung injury [24]. As previously described at ECMO start, ultra-protective ventilation with V_T_ and RR reduction, while PEEP remained constant, decreased mean airway pressure and Crs [25]. This rapid change of Crs can be the consequence of alveolar derecruitment and not only the worsening of lung injury. Patients started vv-ECMO with a lower Crs because of severe lung injury and alveolar derecruitment, and we found that a persistent decrease of Crs during the follow-up is associated with higher mortality.

The follow-up of Crs on the first ten days is relevant as no intensivist would make any prognosis from very early ventilatory data. We determined thresholds of delta Crs, between day one and day five or day ten. However, even if appealing, these thresholds should be interpreted with caution as by performing such analyses, we are at risk of misestimation knowing that three patients died before day ten, that 15 Crs are missing at day one or five and that 35 Crs are missing at day one or ten.

In an international, prospective cohort study of patients undergoing vv-ECMO for non-COVID-19 ARDS, evolution over time of tidal volume (V_T_) and driving pressure (ΔP) (*i.e.,* expression of static respiratory compliance (Crs)), was associated with the outcome [4]. $\Delta$ P and not Crs has been previously reported as a strong predictor associated with mortality in ARDS patients supported with vv-ECMO [26, 27]. In non-COVID-19 patients, Schmidt et al*.* showed that $\Delta$ P was an independent risk factors of 180-day mortality. However, $\Delta$ P was collected at non-successive times on the whole ICU stay. By contrast, in the present study we collected all respiratory variables from day one to ten to approach closely the respiratory system mechanics at the bedside. Moreover, we did not include $\Delta$ P in our model as ventilatory settings aimed to target this variable below 15 cmH_2_O.

The increase in sweep gas flow over time is also a risk factor of 180-day mortality. This was not the consequence of membrane dysfunction over time, but an increase of lung injury with more dead space and the inability of the lung to eliminate CO_2_ and thus the need to maintain an extracorporeal CO_2_ removal that may be a witness of evolution towards fixed lung fibrosis [28].

Finally, importance of pre-existent lung disease should be also underlined. This factor was not found in first COVID-19 series of patients invasively ventilated with or without vv-ECMO as a risk factor of death [6, 29, 30]. One possible explanation was that, in these studies, patients were majorly included during the first wave of the pandemic during which ICU-bed resource was scarce [31, 32]. A more drastic patient selection might be suspected.

Our study has limitations. First, it is a retrospective study with its inherent limitations. However, missing data are scarce, and we used a proven statistical method accounting time-dependent variables on the outcome to overcome this limit [4]. Second, ventilatory management may have influenced patient’s outcome. However, due to its retrospective nature, several ventilatory modes were prescribed over time in a same patient. The first ten days, only 25% had no change (23 patients), while 25% had more than three ventilatory modes prescribed (*n* = 15) which did not allow us to investigate a potential association between ventilatory mode and Crs. Third, patients were included in high-volume centers, all experts in the management of patients requiring vv-ECMO with highly homogenized practices at bedside. Thus, our findings might be not fully representative of the practices in low-volume centers. Fourth, patients with low compliances could have needed more time on ECMO to recover. Indeed, 35 undergone a withdrawal of care. Nevertheless, in this study ECMO duration was twice as long in non-survivors (28 days) as already published COVID and non-COVID ARDS cohorts with ECMO [4, 6, 33]. The decision of withdrawal of care, when there was no likely favorable outcome, was multifactorial and based on a consensus within the team in charge. However, despite an established protocol, we could not exclude that withdrawal decisions may have been influenced by resource issues, specifically in the very beginning of the pandemic. Fifth, the successive pandemic waves and the associated-resource issues can have influenced the outcome of patients. Indeed, during the first wave regions with the highest burden of care in ICU were associated with up to 2.2-fold increase of death rate [31]. We handled this by including “waves of pandemic” as a confounding variable in the multivariate Cox model. This variable was not independently associated with the 180-outcome. Moreover, in this study, 81% of patients were included after the first wave. Finally, if early ventilatory parameters might provide some hints to determine the evolution of CARDS patients supported by vv-ECMO, in many cases, only changes over a prolonged time will be decisive and our finding cannot be extrapolated beyond CARDS patients.

## Conclusions

In this cohort study of patients with CARDS supported by vv-ECMO, with a $\Delta$ P targeted below 15 cmH_2_O and a stable PEEP, Crs evolution measured during the first ten days following vv-ECMO implantation might be a prognostic factor independently associated with 180-day survival status. Thus, intensivists should pay attention to the first ten days Crs since its value may provide information on the patient’s prognosis. These data apply only to patients with CARDS supported by vv-ECMO and should be interpreted with caution as withdrawal decisions may have been influenced by resource issues in the very beginning of the pandemic.

## Supplementary Information

**Additional file 1: Figure S1.** Missing values inspection for the variables included in the final multivariate Cox model. CRS: Static respiratory compliance. **Table S1.** Ventilatory characteristics before and after vv-ECMO implantation in patients with available data on the 2 timepoints. **Table S2.** Baseline characteristics according to waves. **Table S3.** univariate Cox models for 180-day mortality with time-fixed and time-dependent variables.

## Data Availability

The datasets used and analyzed during the current study are available from the corresponding author on reasonable request.

## Abbreviations

ARDS: Acute respiratory distress syndrome
BMI: Body mass index
CARDS: COVID-19-associated ARDS
Crs: Static respiratory compliance
ICU: Intensive care unit
MV: Mechanical ventilation
PEEP: Positive end-expiratory pressure
P_high_: High pressure
P_low_: Low pressure
P_plat_: Plateau pressure
RR: Respiratory rate
V_T_: Tidal volume
vv-ECMO: Veno-venous extra-corporal membrane oxygenation
ΔP: Driving pressure
