# Supplementary material for: Prognostic value of respiratory compliance course on mortality in COVID-19 patients with vv-ECMO
Source: Ann Intensive Care. 2023 Jun 21;13:54. doi: 10.1186/s13613-023-01152-7 (PMC10284783; doi:10.1186/s13613-023-01152-7)
Supplement: Supplementary file 1 — Additional file 1: Figure S1. Missing values inspection for the variables included in the final multivariate Cox model. CRS: Static respiratory compliance. Table S1. Ventilatory characteristics before and after vv-ECMO implantation in patients with available data on the 2 timepoints. Table S2. Baseline characteristics according to waves. Table S3. univariate Cox models for 180-day mortality with time-fixed and time-dependent variables. [file 13613_2023_1152_MOESM1_ESM.docx]

**Prognostic value of respiratory compliance course on mortality in COVID-19 patients with vv-ECMO**

**Additional file**

**METHODS**

vv-ECMO implantation process

vv-ECMO cannulation was performed locally for patients primarily admitted to the ICUs or, for non-transportable patients in peripheric hospital, by mobile ECMO retrieval teams. These teams were available 24 hours a day and 7 days a week. All implantations were percutaneously ultrasonography-guided, with a venous drainage cannula (from 23 to 29 Fr) inserted *via* the femoral vein, and a return cannula (from 17 to 19 Fr) inserted *via* the right jugular vein. Proper positions of cannula tips were checked on a post-implantation chest X-Ray or by ultrasound. Sufficient distance between tips of the cannulas was ensured to avoid excessive recirculation.

Initial management

The PEEP level on ECMO was systematically personalized by different techniques, depending on device availabilities. Three techniques were used for PEEP titration: esophageal pressure, electrical impedance tomography and alveolar opening pressure. This allowed us to identify the optimal PEEP for each patient to obtain the best Crs.

Sedation was managed to target a Richmond Agitation-Sedation Scale score of -4 / -5 assessed every 4 hours. Neuromuscular blocker agents were used systematically during the first 48 hours after vv-ECMO initiation and the decision to pursue them was left at intensivist discretion.

Coagulation management

Continuous infusion of unfractionated heparin was started immediately after vv-ECMO implantation and an anti-Xa at 0.2-0.6 was targeted and checked every day. Transfusion was performed when hemoglobin was lower than 7 g/dl in case of SaO_2_ remained below 90% to optimize oxygen delivery. Platelet transfusions were discouraged except in case of severe bleeding or if platelets were lower than 20 G/l. Fibrinogen level was maintained above 1.5 g/L.

vv-ECMO weaning

Assessment for weaning was performed daily after the first 48h. First, the weaning process was only initiated in patients presenting a significant improvement in Crs defined by a V_T_ at 6-7 mL/kg of ideal body weight with a PEEP lower than 12 cmH_2_O resulted in a P_plat_ lower than 27 cmH_2_O with FiO_2_ lower or equal to 60%. Then, the weaning process started by switching off ECMO sweep gas flow and maintaining a minimum ECMO flow upper than 3L/min. For patients with a body mass index (BMI) greater than 35 kg/m^2^, the P_plat_ target was lower than 35 cmH_2_O. The arterial oxygenation and decarboxylation (generally PaO_2_ greater than 65 – 70 mmHg and PaCO_2_ lower than 45 – 50 mmHg) were checked at three, six and twelve hours during the trial.

*Withdrawal of care management*

ECMO stopping rules were based on clinical and radiological factors: Clinically, a lack of a significant respiratory improvement over time (Crs for instance, persistent hypoxemia or hypercapnia at repeated ECMO weaning trials). Deterioration of the patient’s general condition was also a major factor. When needed, i.e. in most cases, a chest computed tomography was performed to ensure the unfavorable evolution of the parenchymal involvement with radiological signs of pulmonary fibrosis (traction bronchiectasis and/or reticulations). Specifically, in France, the rules of withdrawal are regulated (Law Clays Leonetti N°2016-87). Thus, any withdrawal of care can be decided only after gaining a consensus between medical, paramedical teams and the relatives. Moreover, a second expert opinion, independent from the team in charge must be documented in the record.

Statistical analysis

To determine factors associated with the primary outcome, a time-dependent analysis has been performed. The strength of this specific Cox model relies on the integration of within-individual time variation of the covariate to predict individual response at each time interval when the covariate varies. In other words, in a survival analysis, time-varying covariates are covariates whose value can change during follow-up. In our manuscript, C_RS,_ for instance, is a time-dependent covariate and considering its variation over time improves the accuracy of the prediction compared to a unique value at a specific time. Variables included were chosen on literature and were *a priori* discussed among authors. Thus, the variable “corticosteroids for COVID-19” was not included as no patient received them during the first wave of the pandemic. “PaO_2_/FiO_2_” variable was also not included as a direct consequence of the vv-ECMO settings. “ΔP” as a target variable in the ventilatory management below 15 cmH_2_O and thus “PEEP” and “P_plat_” were also not included. All remaining parameters with a p value <0.1 were included in the multivariate time-dependant model. To build the multivariate time-dependent cox model, a forward stepwise selection of the variables was used. The Null model included Crs time-dependent variable from day one to day ten. Log-linearity for continuous variables, Schoenfeld residuals and variance inflation factors for multicollinearity were checked.

## Figure S1: Missing values inspection for the variables included in the final multivariate cox model. CRS: Static respiratory compliance.


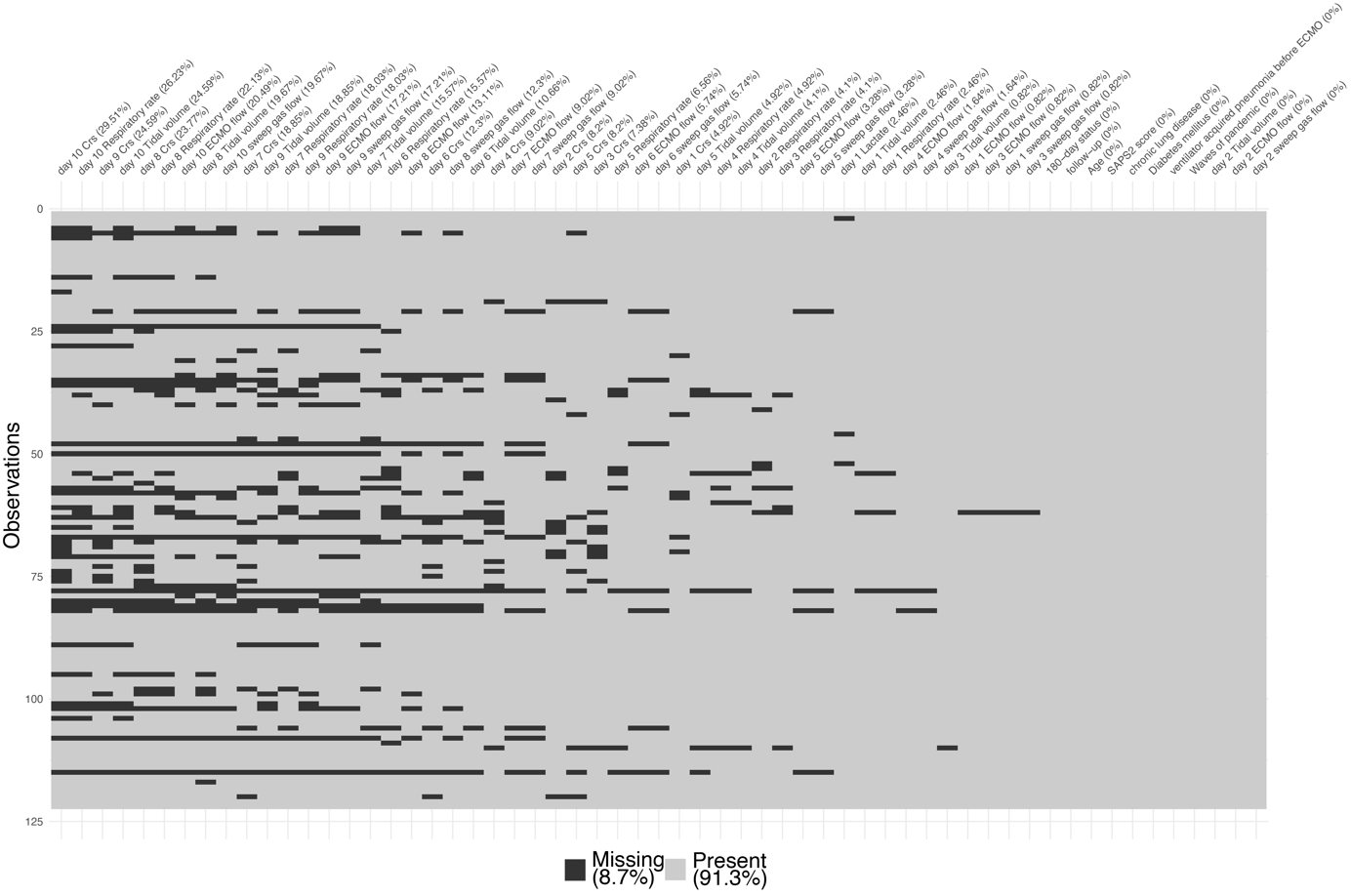


## Table S1: Ventilatory characteristics before and after vv-ECMO implantation in patients with available data on the 2 timepoints

| variable | N available | before ECMO | N available | day 1 after ECMO | p |
| --- | --- | --- | --- | --- | --- |
| Respiratory rate (c/min) | 90 | 28 (26 - 30) | 90 | 12 (10 - 15) | <0.0001 |
| Tidal volume (ml/kg) | 100 | 6 (5 - 6) | 100 | 3 (2 - 4) | <0.0001 |
| Crs (ml/cmH₂O) | 97 | 23.2 (17.9 - 28.6) | 97 | 15.0 (10.0 - 21.4) | <0.0001 |
| Plateau pressure (cmH₂O) | 87 | 28 (25 - 32) | 87 | 25 (22 - 27) | <0.0001 |
| PEEP (cmH₂O) | 104 | 12 (9 - 14) | 104 | 12 (10 - 15) | 0.0116 |
| Driving pressure (cmH₂O) | 87 | 17 (15 - 20) | 87 | 12 (9 - 14) | <0.0001 |

*Values are expressed as median [IQR].*

*ECMO: extracorporeal membrane oxygenation; PEEP: positive end-expiratory pressure; srC: static respiratory compliance.*

## Table S2: Baseline characteristics according to waves

| **variable** | **N** | **Global population** | **N** | **Wave 1** | **N** | **Wave 2** | **N** | **Wave 3** | **N** | **Wave 4** | **p** |
| --- | --- | --- | --- | --- | --- | --- | --- | --- | --- | --- | --- |
| **Demographics** |  |  |  |  |  |  |  |  |  |  |  |
| Age (years) | 122 | 59 (52 - 64) | 23 | 54 (46 - 62) | 25 | 63 (61 - 66) | 53 | 58 (50 - 64) | 21 | 58 (51 - 62) | 0.003 |
| Male gender | 122 | 83 (68 %) | 23 | 19 (83 %) | 25 | 18 (72 %) | 53 | 34 (64 %) | 21 | 12 (57 %) | 0.27 |
| Body mass index (Kg/m²) | 122 | 33 (28 - 37) | 23 | 33 (28 - 36) | 25 | 32 (26 - 37) | 53 | 32 (29 - 36) | 21 | 34 (31 - 39) | 0.38 |
| **Medical History** |  |  |  |  |  |  |  |  |  |  |  |
| Hypertension | 122 | 60 (49 %) | 23 | 7 (30 %) | 25 | 14 (56 %) | 53 | 25 (47 %) | 21 | 14 (67 %) | 0.096 |
| Diabetes mellitus | 122 | 30 (25 %) | 23 | 7 (30 %) | 25 | 4 (16 %) | 53 | 10 (19 %) | 21 | 9 (43 %) | 0.11 |
| Cardiac disease | 122 | 11 (9 %) | 23 | 3 (13 %) | 25 | 1 (4 %) | 53 | 6 (11 %) | 21 | 1 (5 %) | 0.65 |
| Lung disease | 122 | 29 (24 %) | 23 | 3 (13 %) | 25 | 6 (24 %) | 53 | 15 (28 %) | 21 | 5 (24 %) | 0.57 |
| Renal disease | 122 | 10 (8 %) | 23 | 0 (0 %) | 25 | 2 (8 %) | 53 | 4 (8 %) | 21 | 4 (19 %) | 0.14 |
| Immunosuppression | 122 | 15 (12 %) | 23 | 2 (9 %) | 25 | 2 (8 %) | 53 | 7 (13 %) | 21 | 4 (19 %) | 0.69 |
| **Pre-ECMO mamagement** | |  |  |  |  |  |  |  |  |  |  |
| SAPS2 score | 122 | 43 (34 - 55) | 23 | 44 (34 - 62) | 25 | 41 (32 - 51) | 53 | 41 (34 - 46) | 21 | 55 (40 - 63) | 0.100 |
| Delay between first symptoms to ECMO (days) | 109 | 16 (10 - 21) | 22 | 9 (7 - 14) | 20 | 18 (14 - 24) | 49 | 17 (12 - 21) | 18 | 14 (8 - 24) | 0.005 |
| Mechanical ventilation duration before ECMO (days) | 122 | 4 (1 - 9) | 23 | 4 (2 - 7) | 25 | 8 (2 - 11) | 53 | 4 (1 - 9) | 21 | 3 (1 - 7) | 0.11 |
| Corticosteroids for COVID-19 | 122 | 85 (70 %) | 23 | 1 (4 %) | 25 | 18 (72 %) | 53 | 48 (91 %) | 21 | 18 (86 %) | <0.0001 |
| Tocilizumab for COVID-19 | 122 | 16 (13 %) | 23 | 2 (9 %) | 25 | 3 (12 %) | 53 | 8 (15 %) | 21 | 3 (14 %) | 0.94 |
| Prone positionning | 122 | 117 (96 %) | 23 | 23 (100 %) | 25 | 24 (96 %) | 53 | 50 (94 %) | 21 | 20 (95 %) | 0.85 |
| Mobile ECMO assistance | 122 | 67 (55 %) | 23 | 8 (35 %) | 25 | 19 (76 %) | 53 | 27 (51 %) | 21 | 13 (62 %) | 0.029 |
| Ventilator acquired pneumonia | 122 | 72 (59 %) | 23 | 9 (39 %) | 25 | 20 (80 %) | 53 | 27 (51 %) | 21 | 16 (76 %) | 0.006 |
| **day 1 parameters** | | |  |  |  |  |  |  |  |  |  |
| Lactate (mmol/l) | 119 | 1.4 (1.0 - 2.1) | 23 | 1.6 (1.3 - 2.2) | 25 | 1.3 (1.1 - 1.9) | 51 | 1.4 (0.9 - 2.0) | 20 | 1.4 (0.9 - 2.6) | 0.72 |
| PaO₂/iFO₂ | 122 | 134 (93 - 175) | 23 | 125 (90 - 172) | 25 | 111 (73 - 150) | 53 | 133 (99 - 162) | 21 | 156 (125 - 204) | 0.095 |
| Respiratory Rare (c/min) | 119 | 12 (10 - 15) | 22 | 12 (10 - 15) | 23 | 12 (10 - 15) | 53 | 10 (10 - 12) | 21 | 12 (12 - 15) | 0.17 |
| Tidal volume (ml/kg) | 119 | 2.8 (2.0 - 3.5) | 22 | 3.1 (2.5 - 3.9) | 23 | 2.2 (1.8 - 2.8) | 53 | 2.5 (1.9 - 3.3) | 21 | 3.7 (2.0 - 3.9) | 0.009 |
| Crs (ml/cmH₂O) | 116 | 15 (10 - 22) | 19 | 20 (15 - 29) | 25 | 13 (10 - 20) | 51 | 13 (9 - 21) | 21 | 18 (11 - 27) | 0.038 |
| Plateau pressure (cmH₂O) | 114 | 24 (22 - 26) | 18 | 24 (21 - 26) | 25 | 24 (22 - 25) | 50 | 25 (22 - 27) | 21 | 24 (23 - 26) | 0.90 |
| PEEP (cmH₂O) | 121 | 12 (10 - 15) | 22 | 14 (12 - 15) | 25 | 12 (10 - 14) | 53 | 12 (10 - 14) | 21 | 12 (10 - 15) | 0.20 |
| Driving pressure (cmH₂O) | 114 | 12 (10 - 15) | 18 | 11 (10 - 13) | 25 | 13 (10 - 15) | 50 | 12 (10 - 14) | 21 | 11 (10 - 15) | 0.69 |
| Early right ventricular failure | 122 | 9 (7 %) | 23 | 1 (4 %) | 25 | 2 (8 %) | 53 | 5 (9 %) | 21 | 1 (5 %) | 0.93 |
| **Outcomes** |  |  |  |  |  |  |  |  |  |  |  |
| Steroid for prolonged ARDS | 122 | 82 (67 %) | 23 | 9 (39 %) | 25 | 21 (84 %) | 53 | 40 (75 %) | 21 | 12 (57 %) | 0.003 |
| Vasopressors (day one to day ten, yes/no)) | 122 | 83 (68 %) | 23 | 15 (65 %) | 25 | 20 (80 %) | 53 | 31 (58 %) | 21 | 17 (81 %) | 0.14 |
| Number of days on vasopressors (day one to 10) | 100 | 1 (0 - 4) | 20 | 2 (0 - 3) | 15 | 1 (0 - 3) | 45 | 1 (0 - 3) | 20 | 3 (1 - 4) | 0.20 |
| ECMO duration (days) | 122 | 20 (11 - 30) | 23 | 10 (7 - 16) | 25 | 26 (16 - 36) | 53 | 21 (12 - 30) | 21 | 23 (15 - 29) | 0.0005 |
| Mechanical Ventilation duration (days) | 122 | 33 (23 - 50) | 23 | 25 (18 - 35) | 25 | 44 (26 - 61) | 53 | 34 (24 - 44) | 21 | 34 (28 - 50) | 0.014 |
| in-ICU length of stay (days) | 122 | 38 (27 - 52) | 23 | 34 (21 - 45) | 25 | 45 (28 - 62) | 53 | 38 (28 - 49) | 21 | 36 (28 - 54) | 0.22 |
| 6-month deaths | 122 | 58 (48 %) | 23 | 4 (17 %) | 25 | 16 (64 %) | 53 | 28 (53 %) | 21 | 10 (48 %) | 0.009 |

*Values are expressed as median [IQR] or as number and frequency. n: available data*

*COVID-19: Coronavirus disease 2019; Crs: static respiratory compliance; ECMO: extracorporeal membrane oxygenation; FiO_2_: inspired oxygen fraction; ICU: intensive care unit; PaO_2_: arterial oxygen tension; PEEP: positive end-expiratory pressure; SAPS2: simplified acute physiology*score

According to the French National Institute for Statistics and Economic Studies, the distribution of COVID-19 waves in France was decided as follows:

Wave 1: From March 2, 2020, to July 6, 2020

Wave 2: From July 7, 2020, to January 4, 2021

Wave 3: From January 5, 2021, to July 5, 2021

Wave 4: From July 6, 2021, to December 31, 2021

## Table S3: univariate Cox models for 180-day mortality with time-fixed and time-dependent variables.

| **Variables** | **HR 95%CI** | **p** |
| --- | --- | --- |
| **Demographics and medical history** |  |  |
| *Time-fixed variables* |  |  |
| Age (per 5 years) | 1.4 (1.2-1.6) | 0.00033 |
| Body mass index (per 5 Kg/m^2^) | 0.93 (0.77-1.1) | 0.45 |
| Male gender (%) | 1.3 (0.75-2.4) | 0.34 |
| Hypertension (yes/no) | 1 (0.61-1.7) | 0.93 |
| Diabetes mellitus (yes/no) | 1.6 (0.93-2.8) | 0.086 |
| Cardiac disease (yes/no) | 1.3 (0.55-3) | 0.56 |
| Lung disease (yes/no) | 1.6 (0.93-2.8) | 0.085 |
| Renal disease (yes/no) | 0.75 (0.27-2.1) | 0.58 |
| Immunosuppression (yes/no) | 0.99 (0.45-2.2) | 0.97 |
| **characteristics pre-vvECMO implantation** |  |  |
| *Time-fixed variables* |  |  |
| Delay from first symptoms to ECMO implantation (per 2 days) | 1 (0.98-1) | 0.67 |
| Length of mechanical ventilation before ECMO implantation (per 2 days) | 1.1 (0.97-1.1) | 0.22 |
| Prone positionning (yes/no) | 2.7 (0.38-20) | 0.32 |
| Tocilizumab for COVID (yes/no) | 1.5 (0.76-3) | 0.25 |
| Mobile ECMO assistance (yes/no) | 1 (0.6-1.7) | 0.99 |
| Ventilator acquired pneumonia (yes/no) | 1.9 (1.1-3.3) | 0.028 |
| Shock state (yes/no) | 0.69 (0.41-1.2) | 0.18 |
| Wave of pandemic 1 versus 2-3-4 (yes/no) | 3.81 (1.38-10.54) | 0.01 |
| SAPS2 score (per 5 points) | 1.1 (1-1.2) | 0.027 |
| **characteristics post-vvECMO implantation** |  |  |
| *Time-fixed variables* |  |  |
| Lactate (per 0.5 mmol/l) at day 1 | 1.1 (0.95-1.2) | 0.27 |
| PaO₂/FiO₂ (per 10) at day 1 | 1 (0.97-1) | 0.95 |
| Early right ventricular failure | 0.59 (0.18-1.9) | 0.37 |
| Shock state between day 1 and 10 (yes/no) | 1.5 (0.83-2.7) | 0.18 |
| Shock state: number of days under vasopressor from day 1 to 10 | 1 (0.86-1.3) | 0.67 |
| Delta weight between day 1 to 10 (per 2 kg) | 1.1 (0.97-1.2) | 0.18 |
| *Time-dependent variables* |  |  |
| Crs (per 5 ml/cmH₂O) from day 1 to 10 | 0.59 (0.49-0.71) | <0.0001 |
| Respiratory rate (per 5 c/min) from day 1 to 10 | 0.62 (0.49-0.77) | <0.0001 |
| ECMO flow (per 0.5 L/min) from day 1 to 10 | 1.24 (1.11-1.39) | 0.0001 |
| Sweep gas flow (L/min) from day 1 to 10 | 1.21 (1.12-1.32) | <0.0001 |

*wave 1 was compared to the 3 others

*Crs: static respiratory compliance; ECMO: extracorporeal membrane oxygenation; FiO_2_: inspired oxygen fraction; ICU: intensive care unit; PaO_2_: arterial oxygen tension; PEEP: positive end-expiratory pressure; SAPS2: simplified acute physiology*score
